# An interpretable, clinically grounded framework for digital speech biomarker development in neurodegenerative diseases

**DOI:** 10.3389/fdgth.2026.1794169

**Published:** 2026-04-29

**Authors:** Panying Rong, Lindsey Heidrick

**Affiliations:** 1 Department of Speech-Language-Hearing: Sciences & Disorders, University of Kansas, Lawrence, KS, United States; 2 Department of Hearing and Speech, University of Kansas Medical Center, Kansas City, KS, United States

**Keywords:** digital speech marker, early detection, machine learning, neurodegenerative disease, personalized medicine, phenotyping, progressive communication disorder

## Abstract

**Introduction:**

Communication ability—a key determinant of quality of life—is frequently affected and progressively declines in neurodegenerative diseases. Effective management of progressive communication disorders requires a personalized approach to deliver timely interventions tailored to the evolving profiles of communicative impairment, thereby supporting functional communication throughout the disease course. To this end, reliable tools capable of detecting and quantifying both disease-specific patterns of communicative impairment and within-disease phenotypic variability are urgently needed. This study leverages Artificial Intelligence and advanced data analytics to develop an acoustic-based framework for automated extraction of interpretable, clinically grounded speech markers to enable objective assessment and phenotyping of progressive communication disorders.

**Methods:**

Three groups of participants, including 14 individuals with amyotrophic lateral sclerosis (ALS) and 15 individuals with Parkinson's disease (PD), alongside 10 neurologically healthy controls, performed a standardized oral passage reading task, yielding 739 speech samples. Fifty acoustic features were extracted using an automated analytic pipeline and subsequently clustered into six interpretable composite markers. The clinical utility of these markers was evaluated with the recorded speech samples by examining their (1) associations with standardized metrics of cognitive, motor speech, and overall communicative functions, (2) efficacy for detecting and differentiating disease-specific communicative impairment patterns in ALS and PD using supervised machine learning, and (3) utility for within-disease phenotyping and stratification using unsupervised clustering analysis.

**Results:**

The markers effectively (1) detected subtle subclinical changes across multiple domains prior to substantial declines in functional communication outcomes; (2) differentiated disease-specific patterns of communicative impairment (multiclass area under the curve > 0.90); and (3) identified subgroups with distinct speech profiles within each disease.

**Discussion:**

The findings support the potential of the proposed framework as a clinically translatable, objective tool to facilitate early detection, differential diagnosis, and phenotyping of progressive communication disorders, ultimately advancing personalized, measurement-based care in neurodegenerative diseases.

## 1 Introduction

Neurodegenerative diseases are debilitating, incurable illnesses affecting millions of people worldwide with increasing prevalence in parallel with longer average lifespans (1, 2). The pathophysiology of neurodegenerative diseases is associated with the dynamic propagation of pathological proteins (e.g., TDP-43, Alpha-synuclein, Amyloid-beta, Tau) across neural networks, leading to progressive functional declines in speech, swallowing, cognition, gross and fine motor control, and behavior (3). Progressive loss of speech communication is among the most devastating consequences of neurodegenerative diseases with profound impacts on quality of life (4, 5). Due to the lack of effective disease-modifying treatments, behavioral interventions, including compensatory strategies and exercise-based training, in conjunction with assistive technologies such as voice banking and augmentative and alternative communication (AAC) devices, currently constitute the mainstay of management for progressive communication disorders (6, 7).

To maximize therapeutic effectiveness, a patient-centered approach is advocated, emphasizing the delivery of the right intervention to the right person at the right time to preserve functional communication and quality of life throughout the disease course (6). This approach, broadly referred to as personalized medicine, accentuates two clinical priorities: (1) early detection and referral to support informed decision-making, and (2) systematic longitudinal reevaluations to monitor changes and proactively tailor interventions to patients’ evolving needs (6). To operationalize and translate personalized medicine into clinical practice, there is a critical need for an objective, reliable, and efficient framework to enable early detection, monitoring, and characterization of the complex pathological changes underlying progressive communication disorders.

Pathological changes associated with neurodegeneration initially emerge at subclinical levels during the prodromal phase and progressively advance to overt clinical symptoms and functional declines during the symptomatic phase (8, 9). According to an evolutionary perspective, neural structures and pathways that subserve more recently evolved, higher-order functions—such as communication—are particularly vulnerable to neurodegeneration (10). Consequently, subtle subclinical changes in communicative domains often emerge as early indicators of neurodegeneration in clinically asymptomatic individuals. Identifying and characterizing these changes not only provides the foundation for effective, personalized management of communication disorders, but also offers a lens into the broader pathophysiological picture of neurodegeneration, enabling the development of novel speech-based biomarkers to improve the screening and diagnosis of neurodegenerative diseases.

Existing clinical standards for assessing communication disorders rely primarily on clinician-based symptom evaluations, patient-reported outcomes, and functional assessments (11, 12). These approaches are largely subjective and qualitative, and lack sensitivity to subclinical changes, which provide important mechanistic insights but are often too subtle to detect or are masked by compensatory alterations at the clinical or functional level (8, 9). Recent advances in artificial intelligence (AI), particularly deep learning methods, provide data-driven alternatives to traditional knowledge-based approaches, enabling objective, automatic speech analysis. However, the clinical applications of deep learning methods have been constrained by two major barriers: (1) insufficient large-scale training data, due to practical challenges in clinical data collection, which often results in overfitting and limited generalizability, and (2) a lack of interpretability and transparency inherent to the “black box” nature of deep learning, which undermines patient and clinician trust and hinders the adoption of these methods in routine clinical practice (13). To overcome these barriers, Berisha and Liss (13) propose a methodological shift toward developing explainable and individually validated speech markers, rather than relying on uninterpretable raw data, for AI-based modeling, thereby mitigating the curse of dimensionality that arises from high-dimensional raw data spaces relative to limited training sample sizes. Development of such markers can draw on diverse theoretical and clinical resources within the field of communication sciences and disorders (11, 12, 14), as well as cross-disciplinary resources such as quantitative musicology (15).

Consistent with the methodological recommendations of Berisha and Liss (13), this study seeks to develop a clinically grounded, acoustic-based framework for deriving interpretable speech markers to enable objective assessment and phenotyping of progressive communication disorders. This novel framework aims to facilitate the integration of AI and digital innovations into clinical speech marker development, ultimately advancing personalized care in neurodegenerative diseases. To construct this framework, there are several key methodological considerations. First, the selection of speech tasks should balance ecological validity, diagnostic efficacy, and standardization. We selected a standardized connected speech task—reading of a phonetically balanced passage—that balances ecological validity with standardization, enabling future scaling of the framework to real-world contexts for monitoring disease- and treatment-related changes in everyday communication (16). Second, the selection of speech constructs should account for the complex communicative impairments across cognitive (e.g., memory dysfunction), linguistic (e.g., word finding difficulties), and motor (e.g., muscle weakness) domains (17, 18). Moreover, due to the uncertain etiologies of neurodegenerative diseases, the associated communication disorders are complex and heterogenous in nature, exhibiting both overlapping clinicopathological features across diseases and profound within-disease variability in phenotypic presentations. Therefore, speech constructs should be selected to capture both global disease profiles and individual-specific impairment patterns.

Taking the above considerations into account, the proposed framework integrates multiple analytic algorithms for automated extraction of a wide range of acoustic features to characterize prosody (rhythm, regularity, intonation), complexity (entropy, determinism), temporal organization (articulation rate, pause), and voice quality (dysphonia). These features are theoretically and clinically grounded to inform the underlying motor and/or cognitive-linguistic impairments. Through dimensionality reduction, these features were clustered into six interpretable composite speech markers. The utility of these markers for detecting, differentiating, and phenotyping communication disorders was evaluated in two neurodegenerative diseases: amyotrophic lateral sclerosis (ALS) and Parkinson's disease (PD). These diseases were selected due to the high prevalence of motor speech disorders in both. In ALS, mixed flaccid-spastic dysarthria—reflecting combined upper and lower motor neuron degeneration—is the most common presentation, whereas in PD, hypokinetic dysarthria, resulting from dopaminergic neuron degeneration, constitutes the primary manifestation (11, 12). In addition, cognitive-linguistic impairments, including deficits in executive function, word retrieval, and syntax, frequently co-occur with motor speech impairments in both diseases as a consequence of neuronal degeneration within frontotemporal or fronto-striato-thelamo-cortical networks (19–23).

We hypothesized that the speech markers derived from the proposed framework are capable of (1) capturing subclinical changes across different domains of communicative function in both diseases; (2) accurately detecting and differentiating communication disorders in ALS and PD; and (3) identifying subgroups with distinct patterns of communicative impairment within each disease.

## 2 Materials and methods

The study protocol was approved by the Institutional Review Board of the University of Kansas Medical Center. Written informed consent was obtained from all participants. All study procedures were noninvasive with minimal risk.

### 2.1 Participants

Three groups of participants, including 14 individuals with ALS, 15 individuals with PD, and 10 neurologically healthy controls (HC), enrolled in this study. Participants with ALS and PD were recruited from the neurology clinics at the University of Kansas Medical Center, as well as local therapy and support groups. Given the exploratory nature of this study, an *a priori* sample size design was not performed. Participants were enrolled if they met the following inclusionary criteria: (1) being diagnosed with ALS or PD by a certified neurologist with no other concurrent neurological diseases or injuries; (2) speaking Americal English as the first and primary language; (3) passing a hearing screening at 1,000, 2,000, and 4,000 Hz at 30 dB in the better ear; (4) retaining adequate oral communication capacity, with a speech intelligibility score > 85% (per standardized functional speech assessment as described below); (5) ability to understand instructions and comply with study procedures; and (6) reporting no depression or other psychiatric disorders. Recruitment was restricted to individuals with relatively preserved functional speech profiles, as individuals at this stage are most likely to engage in and benefit from behavioral interventions before their disease has progressed too far to incorporate intervention strategies into daily communication, thereby representing an optimal cohort for behavioral studies.

To evaluate cognitive, motor speech, and global communicative functions, all participants underwent two standardized assessments—the Montreal Cognitive Assessment (MoCA) (24) and the Sentence Intelligibility Test (SIT) (25, 26). MoCA is a brief cognitive assessment tool recommended as part of routine clinical practice for neurodegenerative diseases to evaluate cognitive function and detect impairments that necessitate referral for further neuropsychological evaluations (27). It evaluates all cognitive domains, including executive function, memory, language, visuospatial function, attention, and concentration. The total score on the MoCA was derived as an index of overall cognitive function. SIT is a widely used functional speech assessment, in which participants read 11 randomly generated sentences with increasing length from 5 to 15 words. Speech was audio-recorded and subsequently transcribed and timed by two naïve judges who (1) are native speakers of American English, (2) have normal speech, language, hearing, and cognitive functions, and (3) were unfamiliar with the speaker profiles and speech stimuli. Based on the average responses of the two judges, speech intelligibility was calculated as the percentage of correctly transcribed words to index motor speech function. Additionally, communication efficiency, defined as the number of intelligible words per minute, was derived as a comprehensive index of the overall communicative function (28). The demographic and clinical characteristics of the participants are summarized in Table 1.

**Table 1:** Participants characteristics.

Participant characteristics	ALS (*n* = 14)	PD (*n* = 15)	HC (*n* = 10)	Group comparisons
Demographic characteristics				
Women (N%)	28.57%	40.00%	70.00%	p=.14
Age, years (M;SD)	57.21;14.71	69.87;6.45	66.80;13.02	$\chi^{2}=6.41,\;p=.04$
Clinical characteristics				
Time since diagnosis, years (M;SD)	1.14;1.15	4.36;3.44	n.a.	n.a.
MoCA score (M;SD)	25.36;3.65	24.73;4.20	26.75;1.28	$\chi^{2}=1.34,\;p=.51$
Speech intelligibility, % (M;SD)	96.30;3.37	94.86;3.04	99.45;0.56	$\chi^{2}=18.11,\;p<.001$
Communication efficiency, words per minute (M;SD)	139.17;25.36	156.53;32.61	182.69;23.33	$\chi^{2}=8.10,\;p=.017$

ALS, amyotrophic lateral sclerosis; PD, Parkinson's disease; HC, healthy control; MoCA, Montreal Cognitive Assessment; n.a., not applicable. Group comparisons were conducted using Fisher's Exact Test for sex and Kruskal–Wallis Test for all other variables.

### 2.2 Experimental protocol

**Figure 1:**
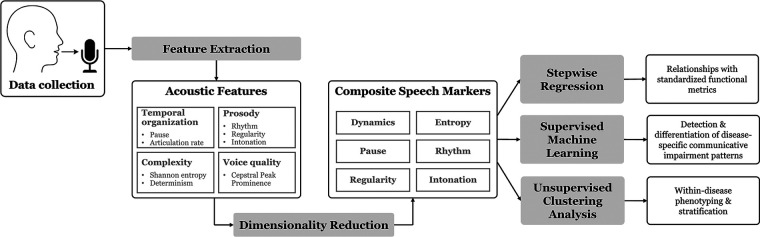
Methodological framework.

The methodological framework is outlined in Figure 1. During data collection, participants read the Rainbow Passage—a standardized, phonetically balanced reading passage consisting of 19 sentences and 330 words—from a monitor, at their habitual speaking rate and loudness. The participants did not have prior experience with this reading passage. Speech was recorded by a head-mounted microphone (dfine 4,188; DPA) placed approximately 5 cm from the left lip corner, pre-processed by a sound conditioner (Xenyx 802; Behringer), and acquired at a sampling rate of 22,050 Hz. Audio gain was set to an appropriate level prior to the experiment and remained fixed throughout the session. Participants with PD were assessed during the “On” state of medication. Using this protocol, a total of 741 speech samples (19 sentences × 39 participants) were acquired from all participants; two samples were discarded due to participant errors, yielding 739 usable samples for the subsequent feature extraction.

### 2.3 Feature extraction

Fifty acoustic features were extracted from each speech sample to characterize temporal organization (articulation rate, pause), prosody (rhythm, regularity, intonation), complexity (entropy, determinism), and voice quality (dysphonia), using an automated data analytic pipeline. These features are summarized in Table 2, and technical details on the feature extraction algorithms are provided below.

**Table 2:** Overview of acoustic features.

Construct	Feature label	Feature interpretation
Temporal organization		
Pause	MeanDur_pause	Mean pause duration
	SdevDur_pause	Standard deviation of pause duration
	CvDur_pause	Coefficient of variation of pause duration
	pct_pause	Percentage of pause duration
Utterance	AR	Articulation rate
Prosody		
Rhythm	hbenvlp_mod_depth_delta_100_300	Rhythmic modulation at prosodic level
	hbenvlp_mod_depth_delta_300_800	
	hbenvlp_mod_depth_delta_1000_3000	
	hbenvlp_mod_depth_delta_3000_8000	
	hbenvlp_mod_depth_theta_100_300	Rhythmic modulation at syllabic level
	hbenvlp_mod_depth_theta_300_800	
	hbenvlp_mod_depth_theta_1000_3000	
	hbenvlp_mod_depth_theta_3000_8000	
	hbenvlp_mod_depth_beta.gamma_100_300	Rhythmic modulation at sub-syllabic level
	hbenvlp_mod_depth_ beta.gamma _300_800	
	hbenvlp_mod_depth_ beta.gamma _1000_3000	
	hbenvlp_mod_depth_ beta.gamma _3000_8000	
Regularity	hbenvlp_PSI_delta_theta_100_300	Regularity of prosodic stress across syllables
	hbenvlp_PSI_delta_theta_300_800	
	hbenvlp_PSI_delta_theta_1000_3000	
	hbenvlp_PSI_delta_theta_3000_8000	
	hbenvlp_PSI_theta_beta.gamma_100_300	Temporal variability of syllable structure
	hbenvlp_PSI_ theta_beta.gamma _300_800	
	hbenvlp_PSI_ theta_beta.gamma _1000_3000	
	hbenvlp_PSI_ theta_beta.gamma _3000_8000	
	density	Overall regularity of acoustic waveform
Intonation	sdevF0	Standard deviation of fundamental frequency
	iqrF0	Interquartile range of fundamental frequency
Complexity		
Entropy	ShanEn_1, ShanEn_2, ShanEn_3, ShanEn_4, ShanEn_5, ShanEn_6, ShanEn_7, ShanEn_8	Shannon entropy of time-frequency representations derived from wavelet packet decomposition
Determinism	DET_dmfcc_1, DET_dmfcc_2, DET_dmfcc_3, DET_dmfcc_4, DET_dmfcc_5, DET_dmfcc_6, DET_dmfcc_7, DET_dmfcc_8, DET_dmfcc_9, DET_dmfcc_10, DET_dmfcc_11, DET_dmfcc_12, DET_dmfcc_13	Determinism of delta mel-frequency cepstral coefficients
Voice quality		
Dysphonia	CPP	Cepstral peak prominence

#### 2.3.1 Temporal organization

The evaluation of temporal organization focused on distinguishing between two types of events during the reading task—utterances and pauses. Four features were extracted to characterize pausing patterns, and articulation rate was calculated to index the speed of utterance production. Pauses were identified using a previously validated algorithm, with baseline parameters set as follows: minimum pause duration = 150 ms, minimum speech duration = 35 ms, minimum signal amplitude threshold = 0.04 (29, 30). Based on the identified pause events, the mean, standard deviation, coefficient of variation (i.e., standard deviation divided by the mean), and total percentage of pause duration were calculated to characterize pausing patterns. Next, all pauses were excluded so that the remaining portions of the task reflected the actual speech articulation time. Articulation rate was then calculated as the number of words produced per unit of articulation time.

#### 2.3.2 Prosody

Within the prosodic domain, 23 features were extracted to characterize rhythm, regularity, and intonation patterns (30–33). Specifically, rhythm was evaluated by examining the modulation patterns of critical-band envelopes at three hierarchically nested, linguistically relevant timescales—delta (0.9–2.5 Hz), theta (2.5–12 Hz), and beta/gamma (12–40 Hz)—corresponding to the rhythms of prosodic, syllabic, and sub-syllabic units, respectively (34, 35). In doing so, the speech acoustic waveform—excluding all pauses—was first decomposed into 28 narrow-band signals spanning evenly along the cochlear frequency map between 100 and 10,000 Hz (36, 37). Next, the Hilbert envelope of each narrow-band signal was down-sampled to 100 Hz and then smoothed using a second-order, zero-lag Butterworth filter with a low-pass cutoff of 40 Hz. To account for individual variability in articulation rate and its effect on rhythmic features, all narrow-band envelopes were normalized via linear time-scaling based on the ratio of each participant's articulation rate to an empirical norm, which was defined as the mean articulation rate of the HC group.

The 28 rate-normalized narrow-band envelopes were aggregated into four critical-band envelopes spanning the following spectral bands: 100–300 Hz, 300–800 Hz, 1,000–3,000 Hz, and 3,000–8,000 Hz. These bands carry distinct and meaningful physiological information: the lowest band (100–300 Hz) reflects source characteristics (e.g., vocal pitch); the intermediate bands (300–800 Hz, 1,000–3,000 Hz) represent articulatory behaviors underlying the first and second formants of vowels; and the highest band (3,000–8,000 Hz) contains articulatory information primarily related to high-frequency consonant noise. The power spectra of these critical-band envelopes were calculated using a 2,048-point Fast Fourier Transform (FFT) with a hamming window. The modulation depth of the spectrum at the delta, theta, and beta/gamma rhythms was then computed as the ratio of spectral power within each target frequency range (i.e., 0.9–2.5 Hz for delta; 2.5–12 Hz for theta; 12–40 Hz for beta/gamma) to the total spectral power. As a result, 12 modulation depth features were derived from the four critical-band envelopes, capturing rhythmic modulation patterns of both voice source and vocal tract configuration across prosodic, syllabic, and sub-syllabic levels.

Beyond the modulation depth at each rhythm, the harmonic coupling between neighboring rhythms was calculated using the phase synchronization index (PSI):

$$\mathrm{PSI}=|e^{i(n\phi_{1}(t)-m\phi_{2}(t))}|$$ (1)

where $\phi_{1}(t)$ and $\phi_{2}(t)$ are instantaneous phases of two oscillatory signals at time *t*; *n* and *m* are integers reflecting the frequency relationship between signals; and $n\phi_{1}(t)-m\phi_{2}(t)$ represents the generalized phase difference. The mathematical operators transform and average the generalized phase difference across time, yielding a single PSI value between 0 and 1, where higher values denote stronger harmonic coupling between the two oscillatory signals.

To calculate PSI, each rate-normalized narrow-band envelope derived above was further band-pass filtered at 0.9–2.5 Hz, 2.5–12 Hz, and 12–40 Hz, using fourth-order, zero-lag Butterworth filters, generating three oscillatory signals that reflect the modulation of the narrow-band envelope at the delta, theta, and beta/gamma rhythms, respectively. These oscillatory signals were then aggregated into four critical-band signals within the 100–300 Hz, 300–800 Hz, 1,000–3,000 Hz, and 3,000–8,000 Hz bands at each target rhythm. PSI was computed between matched critical-band signals at neighboring rhythms using Equation 1, with the coupling ratio n:m set empirically to 2:1 for delta-theta PSI and to 3:1 for theta-beta/gamma PSI (35). These procedures together yielded eight PSI features: the four delta-theta PSI features index the regularity of prosodic stress across syllables, whereas the four theta-beta/gamma PSI features capture intrinsic temporal variability of syllable structure arising from lexical stress and other fine-grained phonological/phonetic factors.

In addition to the PSI features, which capture distinct cues of temporal regularity underlying prosodic and lexical stress patterns within each critical band (e.g., 100–300 Hz for vocal cues; 300–800 Hz and 1,000–3,000 Hz for articulatory cues for vowels), another feature was extracted to characterize the regularity of the integrated acoustic signal by evaluating the “visibility” of the waveform. This concept was adopted from music, where music styles with richer dynamics and more versatile beat patterns (e.g., classical music) are characterized by less homogeneous peaks and, consequently, greater overall visibility of the audio signal (38). Analogously, speech with more versatile stress patterns is expected to generate less regular and more visible peaks in the acoustic waveform, whereas excess-equal stress or reduced stress—both commonly observed in motor speech disorders—tends to diminish the overall peak visibility.

Visibility was evaluated by a graph-theoretical approach adapted from music analysis (38). The speech acoustic waveform was first transformed into a local standard deviation series using Equation 2:

$$V_{j}=\sqrt{\frac{\sum_{(\;j-1)*L+1}^{\;jL}{\left(U_{i}-{\bar{U}}_{j}\right)}^{2}}{L-1}}$$ (2)

where $\left[U_{j}]=[U_{1},\;U_{2},\;\ldots ,\;U_{N}\right]$ is the original signal, $\left[V_{j}]=[V_{1},\;V_{2},\;\ldots ,\;V_{M}\right]$ is the transformed signal, and L is the interval length used for standard deviation calculation, set to 10 ms. Next, the transformed signal was converted into a visibility graph, where individual data points constituted the nodes and internodal connections were determined according to Equation 3:

$$\frac{V_{y}-V_{z}}{y-z}>\frac{V_{y}-V_{x}}{y-x}$$ (3)

If all intermediate nodes $V_{z}$ between two given nodes $V_{x}$ and $V_{y}$ satisfy the condition in Equation 3, $V_{x}$ and $V_{y}$ are connected by an edge. A local peak in the speech signal with great “visibility”—corresponding to a stressed segment—has high connectivity with its neighboring nodes. Based on this notion, a standard graph descriptor, density, which measures the overall level of connectivity, was calculated to quantify global visibility of the signal using Equation 4:

$$\mathrm{density}=\frac{2\times m}{M(M-1)}$$ (4)

where *M* is the total number of nodes, *m* is the total number of edges, and M(M−1) is the largest possible number of edges.

Finally, two features were extracted to characterize intonation patterns. Vocal fundamental frequency was estimated using the cross-correlation method (39), with a moving window of 20 ms for male speakers and of 10 ms for female speakers to account for sex-related differences in vocal pitch. The standard deviation and interquartile range were subsequently computed to index the variability and range of the fundamental frequency contour.

#### 2.3.3 Complexity

Complexity is a multifaceted construct that provides valuable insights into the cognitive-linguistic and/or motor processes underlying speech production. It is most commonly investigated by nonlinear methods such as entropy and recurrence quantification analysis (RQA). Entropy quantifies the information content of a signal (40), whereas RQA evaluates the predictability of behaviors in a dynamical system (41, 42). The speech production system, as a nonlinear dynamical system, generates complex outputs that differ fundamentally from both fully deterministic signals (e.g., sinusoidal waves), which have high predictability but low information content, and completely random signals (e.g., white noise), which are characterized by low predictability and low information content (43). This complexity of speech emerges from both suprasegmental and segmental levels, encompassing features such as prosodic and grammatical intonation, phase-final lengthening, and coarticulation, all of which follow specific linguistic rules but are implemented variably across speakers. These features collectively give rise to a complex speech output that is neither random nor fully predictable. Entropy and RQA provide objective methods for quantifying these aspects of complexity. In pathological speech, suprasegmental and segmental features could be impaired to varying degrees due to cognitive-linguistic and/or motor deficits, resulting in differential changes in complexity (44).

To calculate entropy, the acoustic waveform was decomposed into eight time-frequency representations using a 3-order wavelet packet decomposition (WPD) with the Daubechies wavelet (45). WPD offers a balanced characterization of time–frequency structure, yielding representations that span the entire spectrum, from low-frequency components associated with the formant patterns of voiced sounds to high-frequency components corresponding to noise related to the production of unvoiced sounds. The Shannon entropy of these time-frequency representations was calculated using Equation 5:

$$\mathrm{ShanEn}=-\underset{i}{\sum }s_{i}^{2}\log(s_{i}^{2})$$ (5)

where $s_{i}$ is the i^th^ time-frequency representation. The resulting eight Shannon entropy features quantify the complexity of information content across the entire spectrum.

In addition to spectral complexity, the temporal evolution of the spectrum—reflecting dynamic movements of the vocal tract—is essential for generating smooth and accurate transitions between sounds (e.g., coarticulation) during connected speech. To characterize the complexity of such movements, the first derivatives of the first 13 Mel-Frequency Cepstral Coefficients (MFCCs), also referred to as delta coefficients, were derived as representations of vocal tract movements, analogous to a velocity trace. The first 13 coefficients were selected for analysis, as they are conventionally regarded as capturing linguistically relevant spectral envelope characteristics while minimizing the influence of high-frequency spectral noise in higher-order coefficients. Next, RQA was applied to evaluate the dynamics of these delta-MFCCs based on their recurrence patterns.

Recurrence is defined as the returning of a dynamical system to a previous state in the phase space, in accordance with Equation 6:

$$R_{i,j}=\Theta (\varepsilon -∥{\vec{x}}_{i}-{\vec{x}}_{j}∥)$$ (6)

where ${\vec{x}}_{i}=[u_{i},u_{i+\tau },\ldots u_{i+(m-1)\tau }]$ is a state vector representing the phase space trajectory at the i^th^ sampling point, with embedding dimension *m* and time delay τ; ∥⋅∥ is the Euclidian distance; and Θ is the Heaviside function that equals 1 when $∥{\vec{x}}_{i}-{\vec{x}}_{j}∥\;\leq \;\varepsilon$ and 0 when $∥{\vec{x}}_{i}-{\vec{x}}_{j}∥\;>\varepsilon$. In the recurrent plot, a recurrence point corresponds to a pair of state vectors that are close in the phase space, meeting the following criterion: $∥{\vec{x}}_{i}-{\vec{x}}_{j}∥\;\leq \;\varepsilon$.

Recurrent plots were generated for 1-s, approximately stationary segments of the delta-MFCCs, with parameters set as m=3, τ=5, and ε=0.2 using standard methods (e.g., mutual information, false nearest neighbors). Determinism, defined as the ratio of recurrence points forming diagonal structures to all recurrence points, was calculated for each recurrence plot and then averaged across all plots to quantify the periodic content of the signal. Using this approach, 13 determinism features were extracted from the delta-MFCCs, with higher values indicating greater predictability and lower complexity of the dynamic vocal tract movements.

#### 2.3.4 Voice quality

Voice quality was assessed by cepstral peak prominence (CPP), a recommended acoustic measure of dysphonia that quantifies the relationship between periodic and aperiodic energy in a signal (46). CPP was derived from the cepstrum—the inverse Fourier transform of the logarithmic spectrum—as the height of the most prominent peak (corresponding to the vocal fundamental frequency) relative to a linear regression line fitted to the cepstral envelope. Higher CPP values indicate greater periodicity or harmonicity, whereas lower CPP values reflect increased aperiodic content in the voice signal, typically related to breathy, rough, or hoarse quality.

### 2.4 Feature dimensionality reduction

Since speech production is an integrated act underpinned by a hierarchy of interrelated neurophysiological processes, some of the features derived above are expected to share common variance. To account for such shared variance and reduce the dimensionality of the feature space, the 50 acoustic features were subjected to factor analysis using the *minimal residual factoring* method with *oblimin* rotation (47). This analysis aggregated the features into a lower-dimensional set of factors, where the optimal number of factors was determined by parallel analysis and scree plot. Factor scores were then estimated using the *Ten Burge* method (48). These scores reflect individuals' standing on the latent constructs represented by the factors and can thus be interpreted as composite speech markers, as referenced in the subsequent analyses.

### 2.5 Evaluating the clinical utility of the composite speech markers

The clinical utility of the composite speech markers was evaluated using the speech samples collected from all three participant groups (total *n* = 739). The analyses included: (1) relationships with standardized metrics of cognitive, motor speech, and overall communicative functions; (2) discriminatory efficacy in distinguishing each neurodegenerative disease group from HC and from one another; and (3) data-driven phenotyping and stratification within each disease.

#### 2.5.1 Relationships with standardized functional metrics

The relationships between all composite speech markers and each functional metric, including the MoCA score, speech intelligibility, and communication efficiency, were evaluated using stepwise regression with bidirectional variable selection based on the Akaike Information Criterion (AIC). This stepwise procedure aimed to identify the most significant predictors of each functional metric, thereby informing the relative contributions of individual speech markers to functional declines. In addition, the correlations among the three functional metrics were evaluated using Spearman's ρ to examine the extent to which disease-related changes in these metrics were driven by a common underlying factor—global disease stage/severity—which can confound the interpretation of relationships between the composite speech markers and the cognitive, motor speech, and overall communicative functions captured by these metrics.

#### 2.5.2 Classification

All composite speech markers were fed into multiclass classification models to distinguish the ALS, PD, and HC groups using two supervised machine learning algorithms: support vector machine (SVM) and random forest (RF). SVM is a robust learning technique that allows for flexible mapping of raw data in a high-dimensional space using different kernels to handle complex between-class boundaries in classification problems (49). This study used the nonlinear Radial Basis Function kernel. RF is an ensemble learning method combing individual decision trees trained on randomly selected data samples and features to enhance robustness and reduce overfitting of the model (50). RF also provides a built-in feature importance ranking function, enabling the evaluation of the relative contribution of each speech marker to the classification outcome.

Both classification models were cross-validated through 5-fold cross-validation repeated 10 times. The overall performance of multiclass classification was evaluated using the mean area under the curve (AUC), accuracy, sensitivity, specificity, positive predictive value, and negative predictive value. In addition, the efficacy of the models in distinguishing ALS and PD from HC, as well as from each other, was examined by generating the Receiver Operating Characteristic (ROC) curves for each pairwise classification.

#### 2.5.3 Clustering analysis

To explore the utility of the composite speech markers for within-disease patient phenotyping and stratification, an unsupervised machine learning algorithm—Partitioning Around Medoids (PAM)—was employed to perform clustering analysis on the datasets for the two neurodegenerative diseases. PAM is a robust clustering algorithm through iterative selection of medoids and assigning data points to medoids that minimize the sum of dissimilarities (51). There are two major strengths of PAM: (1) resistance to outliers, generating more stable results for smaller datasets compared to methods such as k-means, and (2) flexibility in handing different data types including both continuous and categorical variables.

Since PAM clustering is based on within-cluster dissimilarity, which could be influenced by disease-unrelated inherent variability in speech production (e.g., age- and sex-related differences), all composite speech markers were adjusted for age and sex to control for their potential confounding effects on the clustering analysis. The resulting adjusted speech markers (continuous variables), together with the clinical diagnosis (a categorical variable with two levels: ALS and PD), were analyzed using the PAM algorithm with the Gower distance metric, which accommodates mixed data types (52), to partition all data samples from the two neurodegenerative diseases into clusters. The number of clusters was determined by the *Gap Statistic* to optimize for within-cluster dissimilarity (53).

Participants were assigned to subgroups based on the clustering of their data samples using a majority rule, whereby the cluster containing the majority of the participant's data samples determined their subgroup. The speech profiles of all identified subgroups were examined by fitting linear mixed-effects models to all age- and sex-adjusted composite speech markers, with subgroup (a categorical variable including all identified disease subgroups and a baseline healthy control group) serving as the fixed effect and a subject-specific intercept included as a random effect. Post-hoc comparisons between each subgroup and the baseline were conducted using estimated marginal means and Cohen's d effect sizes (54).

## 3 Results

### 3.1 Factor analysis

The 50 acoustic features were clustered into six factors, with the rotated factor loadings reported in Supplementary Figure 1. Based on the feature loading patterns, all factors demonstrated interpretable latent constructs: Factor 1 comprised the determinism of all delta-MFCCs and the articulation rate, together reflecting the dynamics (complexity and rate) of vocal tract movements; Factor 2 consisted of all entropy features, representing spectral complexity; Factor 3 included all pause features that jointly characterized pausing patterns; Factor 4 encompassed modulation depth across all three rhythmic bands, with delta-band features loading negatively and theta- and beta/gamma-band features loading positively, together capturing the balance between the modulation of slow prosodic rhythms and fast syllabic and sub-syllabic rhythms; Factor 5 was characterized by density, along with selective PSI features, which jointly reflected regularity; and Factor 6 was composed of the standard deviation and interquartile range of vocal fundamental frequency, representing intonation patterns.

### 3.2 Relationships between composite speech markers and standardized functional metrics

**Figure 2:**
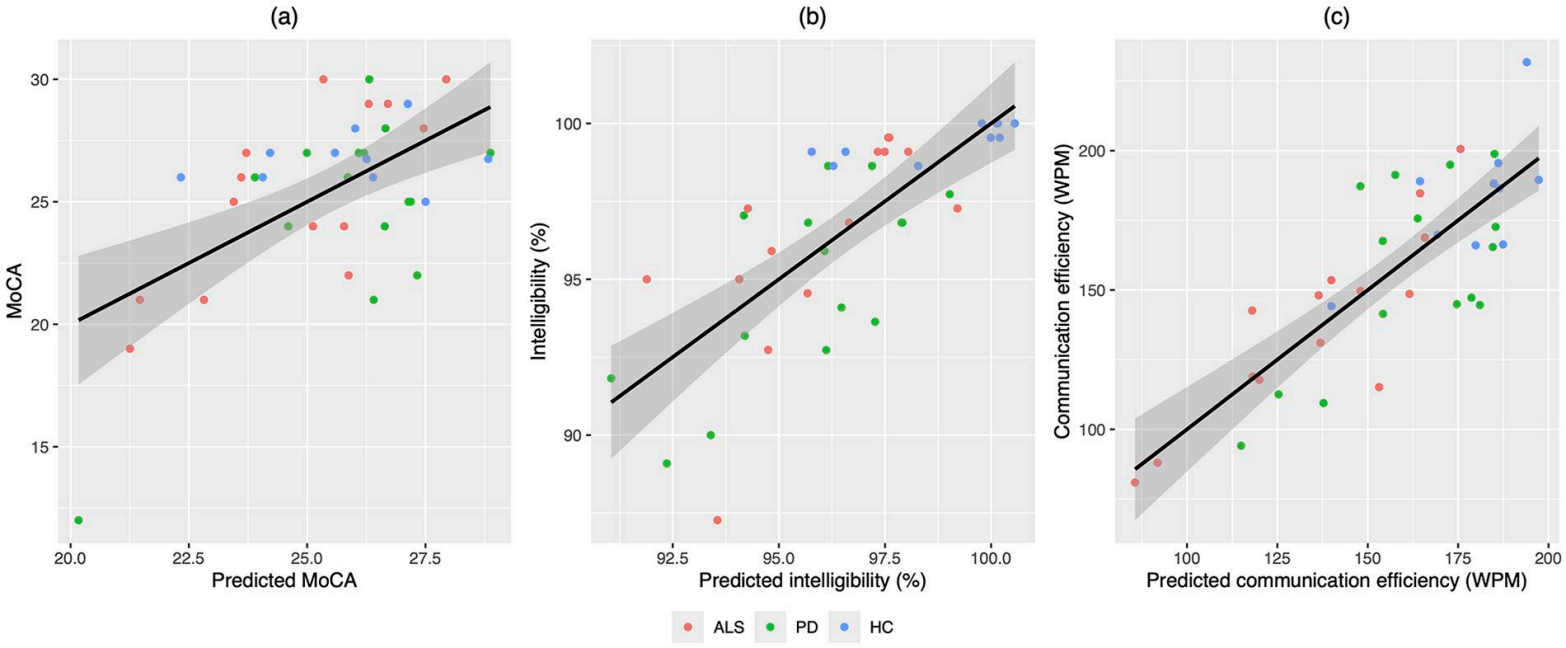
Relationships between standardized functional metrics, including **(a)** MoCA score, **(b)** speech intelligibility, and **(c)** communication efficacy, and outcomes predicted by stepwise regression models. MoCA, Montreal Cognitive Assessment; WPM, word per minute; ALS, amyotrophic lateral sclerosis; PD, Parkinson's disease; HC, healthy control. Solid black lines and the surrounding shaded areas represent the model fits and their confidence intervals estimated by stepwise regression.

As shown in Table 3, the stepwise regressions revealed differential contributions of the composite speech markers to the three functional metrics. Based on the directions of the regression coefficients, declines in the MoCA score were associated with increased pause duration, variability, and proportion (Factor 3), as well as reduced modulation of slow prosodic rhythms accompanied by increased modulation of fast syllabic and sub-syllabic rhythms (Factor 4) (adjusted R^2^ = 0.30). Declines in speech intelligibility were associated with similar changes in pause (Factor 3) and rhythm (Factor 4), in addition to reduced complexity and rate of the dynamic vocal tract movements (Factor 1) and reduced entropy (Factor 2) (adjusted R^2^ = 0.50). Declines in communication efficiency were associated with changes in dynamics (Factor 1), pause (Factor 3) and rhythm (Factor 4), consistent with the correlates of MoCA score and intelligibility, as well as reduced intonation (Factor 6) (adjusted R^2^ = 0.62). The relationships between all functional metrics and the outcomes predicted by stepwise regressions are displayed in Figure 2. Among the three functional metrics, the MoCA score and intelligibility showed no significant correlation (ρ=0.29,p=.071), whereas communication efficiency was significantly associated with both MoCA score (ρ=0.45,p=.0043) and intelligibility (ρ=0.55,p<.001).

**Table 3:** Relationships between composite speech markers (X1—X6) and standardized functional metrics estimated by stepwise regression.

Composite speech marker	MoCA		Speech intelligibility		Communication efficiency	
	Coef	*p*	Coef	*p*	Coef	*p*
X1: Dynamics			−3.94	<.001	−5.47	<.001
X2: Entropy			3.55	.0011		
X3: Pause	−2.53	.016	−2.07	.046	−3.57	.0011
X4: Rhythm	−3.34	.002	−1.61	.12	−2.29	.029
X5: Regularity						
X6: Intonation					1.96	.058

MoCA, Montreal Cognitive Assessment; coef, standardized regression coefficient; p, *p*-value.

### 3.3 Classification

**Figure 3:**
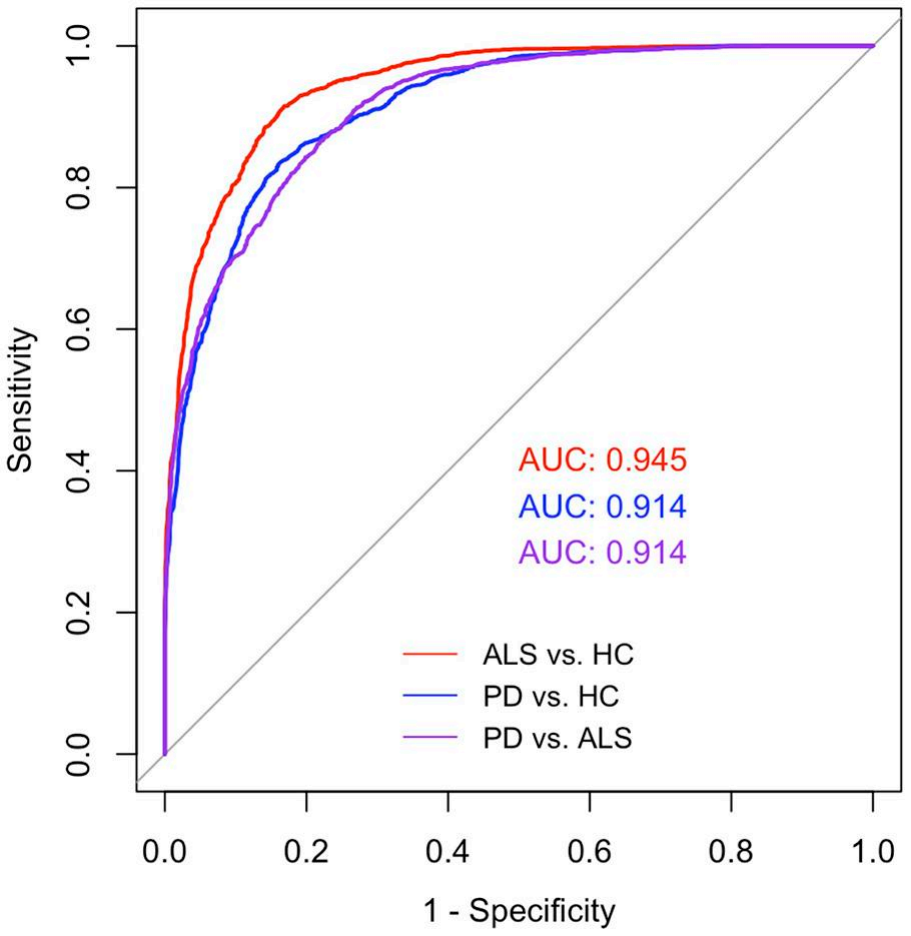
Receiver operating characteristic (ROC) curves for pairwise classifications among amyotrophic lateral sclerosis (ALS), Parkinson's disease (PD), and healthy control (HC) groups, estimated using the random forest algorithm (the support vector machine algorithm exhibited similar performance and was thus not displayed). The area under the curve (AUC) for distinguishing ALS from HC, PD from HC, and PD from ALS is shown in red, blue, and purple, respectively.

The results of multiclass classification are reported in Table 4. Both machine learning algorithms exhibited consistent performance. Based on the feature importance ranking, the composite speech markers, in descending orders of contribution, were entropy (Factor 2), intonation (Factor 6), dynamics (Factor 1), pause (Factor 3), regularity (Factor 5), and rhythm (Factor 4). The ROC curves for pairwise classifications based on the RF algorithm are shown in Figure 3 as an illustrative example. The high AUC values (> 0.90) indicated that the combination of the composite speech markers effectively detected and distinguished disease-specific communicative impairment patterns in ALS and PD.

**Table 4:** Mean performance of multiclass classification models for distinguishing amyotrophic lateral sclerosis (ALS), Parkinson's disease (PD), and healthy control (HC) groups, estimated using the support vector machine (SVM) and random forest (RF) algorithms.

Algorithm	AUC	Accuracy	Sensitivity	Specificity	PPV	NPV
SVM	0.92	0.79	0.78	0.89	0.80	0.89
RF	0.92	0.76	0.76	0.88	0.77	0.88

AUC, area under the curve; PPV, positive predictive value; NPV, negative predictive value.

### 3.4 Clustering analysis

**Figure 4:**
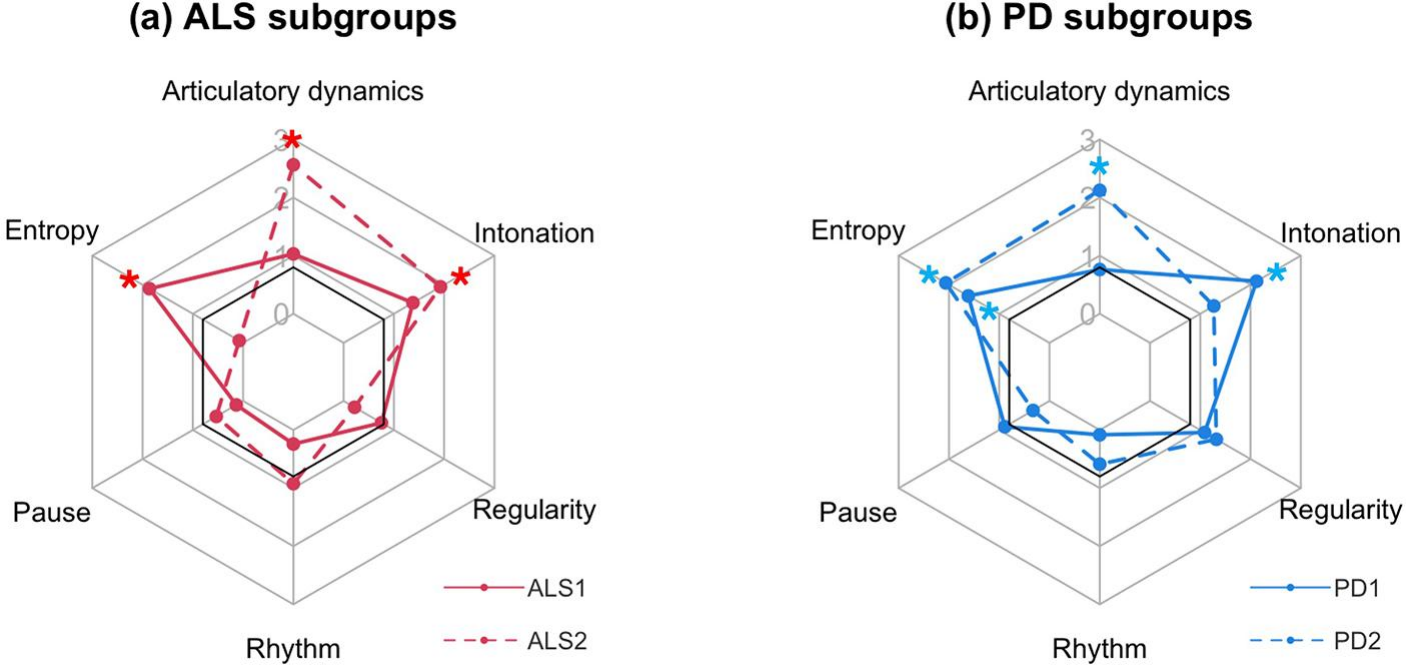
Cohen's d effect sizes for age- and sex-adjusted composite speech markers comparing subgroups of individuals with **(a)** amyotrophic lateral sclerosis (ALS) and **(b)** Parkinson's disease (PD) to the healthy control (HC)-derived baseline (n_ALS1_ = 5; n_ALS2_ = 9; n_PD1_ = 8; n_PD2_ = 7; n_HC_ = 10). Significant effects after false discovery rate adjustment are marked by asterisks.

The PAM algorithm stratified all participants into two ALS subgroups and two PD subgroups. The comparisons of age- and sex-adjusted composite speech markers between each subgroup and the baseline are displayed in the radar charts in Figure 4. Based on the effect sizes and significance for these comparisons, the four subgroups exhibited distinct speech profiles. Among participants with ALS, the first subgroup (*n* = 5) presented primarily with reduced entropy, whereas the second subgroup (*n* = 9) was characterized by impaired dynamics (i.e., reduced complexity and rate) and reduced intonation. The two PD subgroups shared a common feature of reduced entropy but differed in two other markers, with the first subgroup (*n* = 8) primarily exhibiting reduced intonation and the second subgroup (*n* = 7) predominantly characterized by impaired dynamics. Taken together, the dynamics, entropy, and intonation of speech comprised the most informative markers for within-disease stratification.

## 4 Discussion

This study proposes a clinically translatable methodological framework for automated extraction of interpretable, clinically grounded, acoustic-based speech markers to support objective assessment and phenotyping of progressive communication disorders in two neurodegenerative disease cohorts: ALS and PD. These markers were shown to (1) detect and distinguish disease-specific patterns of communicative impairment in ALS and PD, and (2) identify subgroups within each disease. The proposed framework demonstrates strong potential as an efficient tool for integration into routine clinical practice to enhance early and differential diagnosis and to enable more precise phenotyping of progressive communication disorders, thereby facilitating more timely and individualized interventions.

### 4.1 Acoustic-based speech markers capture subclinical changes in the speech production system

Acoustic signals, as the integrated output of the speech production system, are underpinned by a wide range of neurophysiological processes spanning cognitive, linguistic, and motor domains. Hence, from a methodological standpoint, acoustic-based speech markers have the capacity to detect communicative impairments across these interacting domains. Consistent with this theoretical assumption, we identified two such markers—reflecting pausing and rhythmic patterns—that were simultaneously correlated with the MoCA score, speech intelligibility, and communication efficiency (Table 3). Although all functional metrics are influenced by a common underlying factor—global disease stage/severity—the absence of a significant correlation between the MoCA score and intelligibility suggests that disease-related changes in these metrics were not entirely driven by severity-related effects. Accordingly, the observed associations of pausing and rhythmic patterns with the MoCA score and intelligibility are likely to reflect, at least in part, intrinsic linkages to cognitive and motor speech functions beyond those attributable to overall disease severity.

Cognitive-linguistic impairments can impact speech pausing patterns through multiple mechanisms. First, deficits in word retrieval, particularly low-frequency words, can slow word-level motor planning and preparation, resulting in more frequent and longer pauses surrounding the word boundaries (55–57). Second, syntactic deficits could disrupt motor planning and preparation at the phrase level, leading to increased pauses at grammatically inappropriate locations (58–60). Additionally, visuospatial deficits can impair eye-tracking and visuomotor integration and reduce the efficiency of forward planning during reading, further exacerbating pausing disruptions at both word and phrase levels (61, 62). Within the motor speech system, more frequent, longer, and more variable pauses have been associated with respiratory impairment (29), a common motor deficit in both ALS and PD (60, 63, 64). Collectively, these cognitive-linguistic and motor deficits may be captured by the pause-based marker.

Rhythm represents the hierarchical temporal organization of speech at prosodic, syllabic, and sub-syllabic (phoneme) levels (65, 66). In a stress-timed language such as English, the modulation of slow prosodic rhythms serves as the foundation for speech timing, providing a scaffold for fast syllabic and sub-syllabic rhythms to generate precisely timed prosodic stress while allowing more flexible timing at syllabic and sub-syllabic levels (67, 68). The rhythm-based marker quantifies the balance between modulation of slow prosodic rhythms and that of fast syllabic and sub-syllabic rhythms. This marker can be impacted by several cognitive, linguistic, and motor factors. Specifically, syntactic deficits could impair the planning of prosodic phrases in accordance with grammatical structures, resulting in prosodic-level rhythmic disturbances, such as short prosodic phrases, which constrain the temporal window for generating duration contrasts—the most powerful cue for prosodic stress—between stressed and unstressed units (69, 70). Motor deficits—in particular the weakness and rigidity of articulatory muscles—can alter speech timing at syllabic and sub-syllabic levels, leading to prolonged syllables and phonemes that often sound “fragmented” with limited prosodic accentuation (71, 72). These opposing temporal changes at prosodic vs. syllabic and sub-syllabic levels may manifest as a shift in hierarchical rhythmic modulation, characterized by reduced higher-level prosodic modulation accompanied by increased lower-level modulation of syllables and sub-syllabic units, as captured by the rhythm-based marker.

Beyond pausing and rhythmic patterns, two additional markers—reflecting the dynamics and entropy of speech—were found to be correlated with speech intelligibility, indicating their linkages to motor speech function. Impairments in dynamic speech movements, characterized by reduced complexity and rate, are in alignment with two common motor speech deficits—impaired coarticulation and slowness of articulatory movement (73–79). Impaired coarticulation is evidenced by well-documented reductions in formant transitions and consonant clusters (80–83), whereas slowness is linked to muscle weakness (e.g., in ALS) or rigidity/bradykinesia (e.g., in PD). Reduced entropy may reflect an overall decrease in phonemic contrasts, resulting from the degradation of both lower-frequency spectral signatures for voiced sounds and higher-frequency signatures for voiceless sounds (81, 84, 85). Accordingly, the observed association between entropy and speech intelligibility is consistent with the well-established linkage between reduced phonemic contrast and intelligibility decline (81, 84, 85).

The majority of the correlates of communication efficiency overlapped with those of speech intelligibility and MoCA score. This is expected given that communication efficiency is an index of global communicative function, which is presumably influenced by all underlying cognitive, linguistic, and motor processes. This presumption is supported by the significant correlations observed between communication efficiency and both the MoCA score and intelligibility. Only one marker—representing intonation patterns—was identified as a unique correlate of communication efficiency. Although intonation, as a suprasegmental feature, has a less direct impact on the intelligibility of individual words compared with segmental features, it provides important cues that help listeners parse the incoming sound stream into syntactically meaningful units, thereby facilitating auditory comprehension (86–89). Compromise of these intonational cues may increase the cognitive load for parsing and interpreting connected speech, consequently reducing communication efficiency.

Taken together, five of the six composite speech markers were correlated with one or more standardized functional metrics. Only one marker—reflecting regularity—was not selected by the stepwise regression as a predictor of any of the functional metrics. This might be attributed to a task-related effect: the passage reading task may not sufficiently challenge the speech production system in generating contrastive stress, making it less susceptible to stress-related deficits in temporal regularity and their impact on the communication outcomes. Tasks that involve greater natural stress variation and contrasts, such as conversational speech, might be more effective in detecting such deficits. Nevertheless, the observed correlations between the majority of composite speech markers and standardized functional metrics support the validity of these markers as objective indicators of meaningful subclinical changes in the speech production system in both ALS and PD. These changes likely arise from the interaction between cognitive-linguistic and motor domains underlying speech production.

### 4.2 Utility of acoustic-based speech markers for detecting and distinguishing communication disorders in neurodegenerative diseases

As shown in Figure 3, the combination of all acoustic-based markers effectively differentiated both ALS and PD groups from HCs, with an AUC greater than 0.90. By comparison, conventional clinical diagnostic methods exhibit misdiagnosis rates ranging from 13% to 68.4% for ALS and from 15% to 35% for PD (90–92). Given that all participants with neurodegenerative diseases in this study had relatively preserved functional speech profiles (mean intelligibility >94%), the strong classification performance supports the potential of the acoustic-based markers as early indicators of subtle subclinical communicative impairments prior to substantial declines in functional outcomes. These markers may complement existing clinical standards to improve the early detection of progressive communication disorders and also support broader diagnosis of neurodegenerative diseases.

The classic classification system for motor speech disorders relies on clusters of auditory-perceptual features to distinguish different types of dysarthria (11, 12). Within this framework, multiple speech dimensions have been identified that are affected in both ALS and PD but manifest disease-specific profiles, characterized by distinct clusters and relative weights of auditory-perceptual features. These dimensions, including articulation (e.g., imprecise consonants and/or distorted vowels), rate (e.g., slow or variable rate), intonation (e.g., monopitch), pause (e.g., inappropriate silences), stress (e.g., excess-equal or reduced stress), and rhythm (e.g., short phrases or rushes of speech), closely align with the acoustic-based markers derived in this study. The clusters of auditory-perceptual features along these dimensions have been designated as “diagnostic clusters” to guide differential diagnosis of dysarthria (11, 12). Consistent with this framework, the pairwise classification results in this study provide converging evidence for the utility of the acoustic-based markers along these dimensions for distinguishing communication disorders in ALS and PD, supporting their potential as objective complements to traditional clinical approaches for enhanced differential diagnosis.

### 4.3 Utility of acoustic-based speech markers for within-disease patient phenotyping and stratification

Neurodegenerative diseases are characterized by profound heterogeneity in clinical manifestation, progression rate, and underlying pathology (93–95). Such heterogeneity may give rise to subgroups with distinct clinicopathological profiles within a disease. This study employed an exploratory, data-driven approach to phenotype communicative impairments in two neurodegenerative disease cohorts using acoustic-based markers and identified two subgroups within each cohort that exhibited distinct patterns of impairment. The identification of these subgroups has important implications for tailored management of communication disorders.

Among individuals with ALS, the first subgroup presented primarily with reduced entropy, reflecting decreased spectral complexity and, consequently, diminished phonemic contrasts. From a management standpoint, these individuals may benefit from a behavioral intervention known as “clear speech”, which uses hyper-articulation strategies to improve the clarity and precision of sound production (33, 96, 97). The second subgroup showed a more diffuse pattern of impairment in both dynamics and intonation patterns, indicating the involvement of both articulatory and respiratory-laryngeal subsystems. These individuals are likely to benefit from a combination of interventions that target different problems, including (1) volitional rate reduction (also known as “slow speech”), which can extend the temporal window available to weak articulatory muscles for generating complex dynamic behaviors (25, 26, 33, 97, 98), and (2) breath group management, which segments longer utterances into shorter chunks (breath groups), thereby allowing greater respiratory support to modulate intonation within each breath group (99, 100).

Among individuals with PD, the first subgroup was characterized by decreases in both entropy and intonation, which may be attributed to a global reduction in motor drive for speech production. This pattern of impairment aligns with the hallmark features of hypokinetic dysarthria and may therefore benefit from gold-standard behavioral interventions for PD, such as the Lee Silverman Voice Treatment LOUD (101, 102) and *SPEAK OUT!* (103–105)*.* These interventions focus on enhancing motor output by recalibrating the sensorimotor system or bypassing the impaired automatic motor system through the intentional reshaping of speech. The second subgroup exhibited decreases in entropy and dynamics. In addition to gold-standard interventions, this subgroup may derive further benefits from cueing strategies that use metronomic or rhythmic cues to entrain articulatory movements to a specific tempo, thereby mitigating the effect of the impaired internal timing function of the basal ganglia on the dynamics of speech movements (106, 107).

### 4.4 Limitations and future directions

This study provides promising initial evidence supporting the potential of a clinically translatable methodological framework for objective assessment and phenotyping of communication disorders in ALS and PD. To facilitate the ultimate integration of this framework into clinical practice, several limitations must be considered and addressed in future work.

Given the relatively small sample size, the generalizability of the present findings is contingent on external validation in larger, more heterogeneous datasets in future studies. In particular, the results of the clustering analysis should be interpreted as exploratory due to the limited number of participants within each subgroup. To enhance sample diversity, we recruited participants from multiple sources, including university-affiliated neurology clinics and local therapy and support groups. Consequently, comprehensive clinical information was not available for all participants. Although we systematically evaluated participants' communicative function using standardized tests, other clinical characteristics, such as overall disease stage and severity, and genetic mutations, might indirectly influence communicative function and should be considered in future studies.

One of the greatest clinical challenges in assessing progressive communication disorders is the reliable detection of subtle changes during early stages, when functional impairments are minimal. Accordingly, this study recruited participants with relatively preserved functional speech capacities to evaluate the efficacy of the acoustic-based markers for detecting these early, subtle changes. However, it should be noted that individuals at different stages of progressive communication disorders are likely to exhibit distinct profiles of impairment, and the trajectories of progression may vary substantially across individuals (108, 109). Relatedly, additional subgroups may emerge in cohorts with greater representations of individuals at the moderate-severe stages. The responsiveness of the acoustic-based markers to communicative changes emerging at different stages, both within and across individuals, warrants further investigation.

This study applied sentence-level analyses to a dataset with multiple sentences recorded from each participant. Treating each sentence as an individual observation increased the effective sample size and, consequently, the statistical power of the machine learning models. This approach also allowed the extracted features to be evaluated across diverse speech stimuli such that the resulting models are not restricted to specific linguistic contents, thereby facilitating their generalization to unseen contexts. However, the inclusion of multiple observations per participant introduces potential dependencies within the data and may increase the risk of overfitting. To mitigate this concern and to evaluate the generalizability of the exploratory findings of this study, participant-level validation in a larger, independent cohort is warranted. Moreover, because the Rainbow Passage takes approximately 1–2 min to complete, fatigue may accumulate over the course of reading. Such fatigue effects can interact with disease-related impairments, potentially influencing features such as pause, articulation rate, and intonation during sentences toward the later part of the passage. Future research should examine and control for these fatigue-related confounders.

Another future direction is to extend the acoustic-based framework to additional speech tasks, particularly spontaneous speech, which provides more naturalistic and representative contexts to engage neural networks that support cognitive-linguistic processes underlying daily communication. Task-specific markers derived from such contexts could then be applied and validated across a broader spectrum of etiologies, including Alzheimer's disease, in which communication disorders are primarily driven by higher-order cognitive impairments. The current study identified entropy, dynamics, and intonation as the most informative markers for both differential diagnosis and within-disease phenotyping. This is likely because (1) both ALS and PD are conventionally classified as neuromotor disorders, in which impairments in movement initiation or execution represent core diagnostic features; and (2) passage reading imposes lower cognitive-linguistic demands compared with spontaneous speech, thereby offering a more targeted assessment of motor speech function than of cognitive-linguistic processes. As such, the identified markers may primarily reflect motor deficits within the articulatory and respiratory-laryngeal subsystems. In contrast, for cognitively driven communication disorders, other markers, such pause, rhythm, and regularity, may play more important roles in detecting and characterizing the associated cognitive-linguistic deficits.

## 5 Conclusions

Rapid advances in AI and digital technologies present tremendous opportunities for health professions while challenging established standards and values. In speech-language pathology, increasing attention has been directed toward digital speech marker development and technology-assisted clinical decision-making. To facilitate the integration of these technological advances into clinical practice, this study proposes a novel acoustic-based methodological framework for automated extraction of six interpretable, objective speech markers that closely align with the auditory-perceptual feature clusters within the classic clinical system. Using both supervised and unsupervised machine learning methods, we demonstrated the utility of these markers for early detection, differential diagnosis, and phenotyping of progressive communication disorders in ALS and PD, with potential for extension to broader neurodegenerative disease contexts. Given the minimal equipment requirement for acoustic recording, the fully automated analytic pipeline, and the extraction of clinically grounded and interpretable markers, the proposed framework provides a highly efficient and scalable tool that hold strong potential for integration into routine clinical practice to complement existing clinical standards, ultimately advancing timely and personalized care in neurodegenerative diseases.

## Data Availability

The raw data supporting the conclusions of this study will be made available by the authors upon reasonable request and subject to appropriate data-sharing agreements.
