# Supplementary material for: An interpretable, clinically grounded framework for digital speech biomarker development in neurodegenerative diseases
Source: Front Digit Health. 2026 Apr 29;8:1794169. doi: 10.3389/fdgth.2026.1794169 (PMC13168084; doi:10.3389/fdgth.2026.1794169)

Supplementary Figure 1. Results of factor analysis, with rotated factor loadings shown on the horizontal axis and features shown on the vertical axis. Loadings with absolute values greater than 0.40 are highlighted in red, and the corresponding features are considered the primary components of each factor.

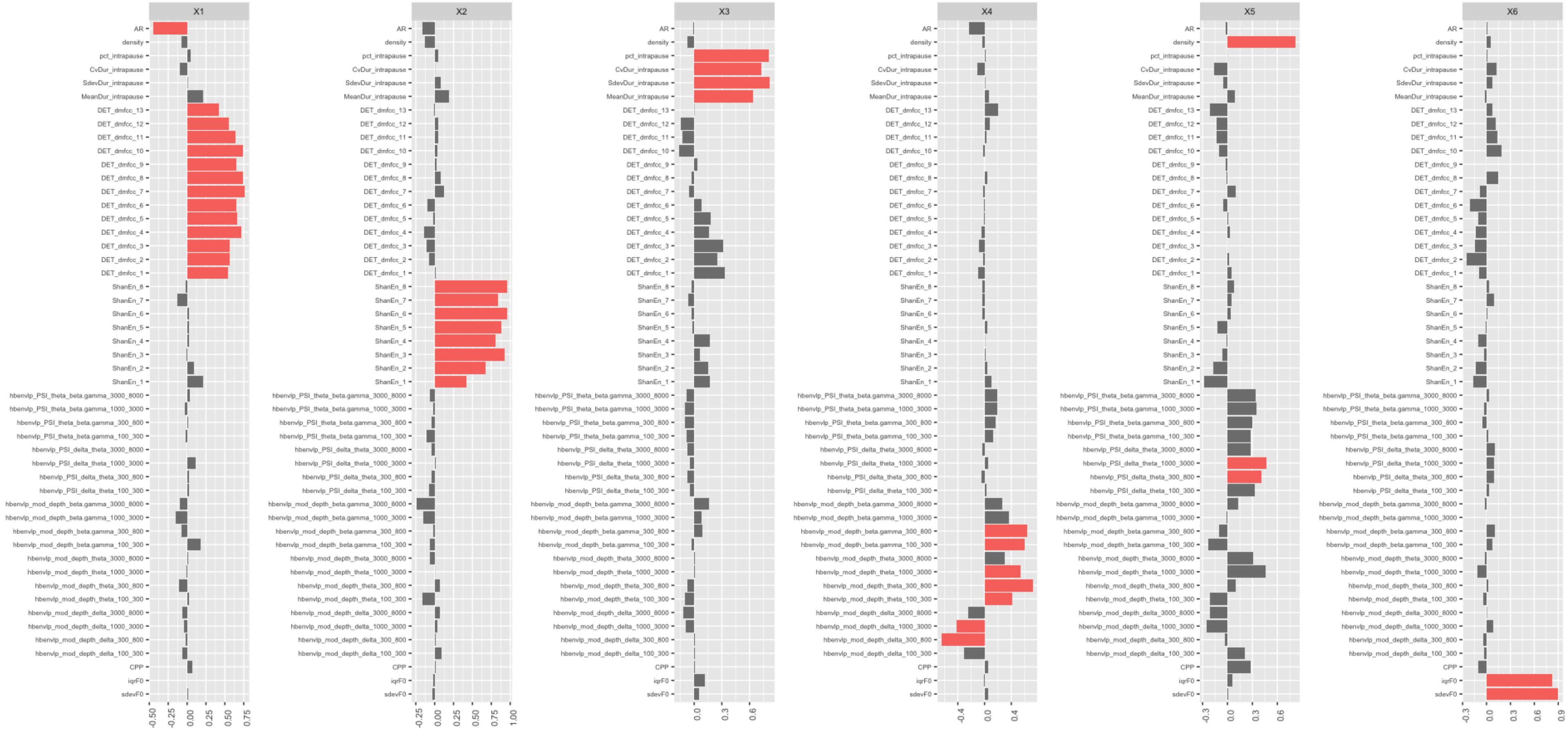

Supplement: Supplementary file 1 [file Image1.pdf]
